# Influence of Short Cationic Lipopeptides with Fatty Acids of Different Chain Lengths on Bacterial Biofilms Formed on Polystyrene and Hydrogel Surfaces

**DOI:** 10.3390/pharmaceutics11100506

**Published:** 2019-10-01

**Authors:** Malgorzata Anna Paduszynska, Magdalena Maciejewska, Damian Neubauer, Krzysztof Golacki, Magdalena Szymukowicz, Marta Bauer, Wojciech Kamysz

**Affiliations:** 1 Department of Inorganic Chemistry, Faculty of Pharmacy, Medical University of Gdansk, 80-416 Gdansk, Poland; maciejewska.kj@gmail.com (M.M.); damian.neubauer@gumed.edu.pl (D.N.); krzysztofgolacki@gmail.com (K.G.); magda.szymukowicz@gmail.com (M.S.); bauerm@gumed.edu.pl (M.B.); kamysz@gumed.edu.pl (W.K.); 2 Pharmaceutical Laboratory Avena Sp. z.o.o., 86-031 Osielsko, Poland

**Keywords:** antimicrobial peptides, lipopeptides, peptide synthesis, bacterial biofilm, biomaterials, biofilm-associated infections

## Abstract

Nowadays, biomaterials are applied in many different branches of medicine. They significantly improve the patients’ comfort and quality of life, but also constitute a significant risk factor for biofilm-associated infections. Currently, intensive research on the development of novel materials resistant to microbial colonization as well as new compounds that are active against biofilms is being carried out. Within this research, antimicrobial peptides (AMPs) and their analogues are being intensively investigated due to their promising antimicrobial activities. The main goal of this study was to synthesize and evaluate the antimicrobial efficacy of short cationic lipopeptides that were designed to imitate the features of AMPs responsible for antimicrobial activities: positive net charge and amphipacity. The positive charge of the molecules results from the presence of basic amino acid residues: arginine and lysine. Amphipacity is provided by the introduction of decanoic, dodecanoic, tetradecanoic, and hexadecanoic acid chains to the molecules. Lipopeptides (C_16_-KR-NH_2_, C_16_-KKK-NH_2_, C_16_-KKC-NH_2_, C_16_-KGK-NH_2_, C_14_-KR-NH_2_, C_14_-KKC-NH_2_, C_12_-KR-NH_2_, C_12_-KKC-NH_2_, and (C_10_)_2_-KKKK-NH_2_) were synthesized using a novel solid-phase temperature-assisted methodology. The minimum inhibitory concentrations (MICs), minimum biofilm eradication concentrations (MBECs), and minimum biofilm formation inhibitory concentrations (MBFICs) were determined for the following bacterial strains: *Staphylococcus aureus* ATCC 25923, *Staphylococcus epidermidis* ATCC 14990, *Enterococcus faecalis* ATCC 29212, *Escherichia coli* ATCC 25922, *Pseudomonas aeruginosa* ATCC 9027, and *Proteus mirabilis* PCM 543. The biofilms were cultured on two types of surfaces: polystyrene plates (PS) and contact lenses (CL). The lipopeptides exhibited the ability to inhibit the growth of bacteria in a liquid medium as well as on the PS and CL. The compounds also eliminated the bacterial biofilm from the surface of both materials. In general, the activity against gram-positive bacteria was stronger in comparison to that against gram-negative strains. There were certain discrepancies between the activity of compounds against the biofilm cultured on PS and CL. This was especially noticeable for staphylococci—the lipopeptides presented much higher activity against biofilm formed on the PS surface. It is worth noting that the obtained MBEC values for lipopeptides were usually only a few times higher than the MICs. The results of the performed experiments suggest that further studies on lipopeptides and their potential application in the treatment and prophylaxis of biofilm-associated infections should be conducted.

## 1. Introduction

The common use of medical devices and implants in modern medicine results in a significant risk of biofilm-associated infections. Regardless of the level of advancement of biomedical implants or tissue-engineering constructs, they constitute a potential surface for microbial colonization [1,2,3,4,5,6]. The number of biofilm-related infection events has increased in recent years. It is estimated that approximately 80% of chronic infections are the result of biofilm formation on the surfaces of applied medical materials, including cardiac implants, catheters, vascular and orthopedic prostheses, or other implants [7]. Biofilm-associated infections are difficult to eradicate and often lead to the complete replacement of biomedical equipment, which is not always effective in preventing the recurrence of infections [8,9,10].

The most common strains that form a biofilm on the surface of biomaterials include Staphylococcus aureus (SA), Staphylococcus epidermidis (SE), Enterococcus faecalis (EF), Streptococcus viridans (SV), Escherichia coli (EC), Klebsiella pneumoniae (KP), Proteus mirabilis (PM), and Pseudomonas aeruginosa (PA) [11,12,13]. In most cases, cardiovascular implant infections are the consequence of the colonization of SA and SE strains [14,15]. EF strains are associated with the development of infections in patients with a central puncture or who are mechanically ventilated [16]. In addition, the bacteria of this species are often isolated in patients with orthopedic implants. The biofilms formed by EF and SV are responsible for endocarditis [17]. The gram-negative microorganisms are the most common etiological factors of urinary tract infections and infections related to the use of urological catheters [18].

Biofilm formation on medical devices is strongly associated with additional mortality and healthcare costs [10]. Despite the constant progress in pharmaceutical sciences, reliable methods of biofilm prevention and treatment are still to be developed. However, promising antibiofilm compounds are antimicrobial peptides (AMPs) and their synthetic analogues.

AMPs are widely distributed in nature as developmental components of the innate immunity of living organisms [19]. The majority of AMPs are cationic, amphipathic molecules that exhibit strong activity against a broad spectrum of pathogens including multi-drug resistant strains. Their ability to eliminate and prevent biofilm structures has been proven in in vitro and in vivo studies [20,21,22,23]. AMPs interact with negatively charged bacteria surfaces, resulting in the destabilization and disruption of cell membranes. Due to their non-specific, rapid mechanism of action, their potential to trigger antimicrobial resistance is significantly lower in comparison to conventional antibiotics. Due to these properties, AMPs are especially attractive for clinical application as an alternative to standard therapy [24,25]. However, there are several significant limitations, such as poor stability and high production costs, that need to be overcome before AMPs can be feasibly introduced for clinical use. A variety of strategies have been proposed in order to remove these obstacles. These include designing shorter analogues or mimics of AMPs with the desired properties.

As a result of previous attempts made to improve biological and physical properties as well as to optimize the production methods of AMPs, short cationic lipopeptides have been developed [26]. The compounds have been designed based on the features of AMPs that are directly associated with antimicrobial activities: amphipathicity and cationic net charge [27,28]. The compounds are composed of a hydrophobic fragment of a fatty acid combined with the peptide chain of positively charged amino acids. It has been shown that acylation of cationic peptides with fatty acid significantly improves their antimicrobial spectrum activity and determines a higher resistance to proteolytic degradation [29,30]. This kind of structure determines the surface-active properties of lipopeptides and enables the interactions with negatively charged microbial membranes, leading to rapid-kill drug-resistant pathogens [31].

Previous research on short cationic lipopeptides confirmed the high activity against clinical isolates of *S. aureus* [32]. C_16_-KK-NH_2_ demonstrated high antistaphylococcal activity against antibiotic resistant strains [33]. Short lipopeptides obtained by Serrano et al. demonstrated high activity against the strains of SA, both MSSA as well as MRSA [34]. Similar activity was demonstrated by lipopeptides composed of tryptophan and ornithine residues combined with capric, caproic, caprylic, lauric, myristic, and palmitic acids [35]. In our previous research, short lipopeptides containing arginine and lysine residues and palmitic acid demonstrated strong antibiofilm activity against clinical strains of SA [36].

The lipopeptides used in this study were inspired by our previous work on lipopeptides that consisted of a few basic amino acid residues (1–4) and fatty acid chains (C8–C16) [37]. Moreover, lipopeptides with two fatty acid chains tested in our previous study revealed promising potential as antimicrobial agents [38]. As a result, a series of lipopeptides with one fatty acid chain (C_16_-KR-NH_2_, C_16_-KKK-NH_2_, C_16_-KGK-NH_2_, C_14_-KR-NH_2_, C_12_-KR-NH_2_) and two fatty acid chains (C_16_-KKC-NH_2_, C_14_-KKC-NH_2_, C_12_-KKC-NH_2_, (C_10_)_2_-KKKK-NH_2_) were chosen for the research. The goal of the present study was to produce the above mentioned short lipopeptides using a novel, temperature-assisted synthesis methodology and to evaluate their antimicrobial properties with regard to their potential use against pre-formed bacterial biofilms and biofilm formation.

## 2. Materials and Methods

### 2.1. Peptide Synthesis

Lipopeptides 1–8 (Table 1) were synthesized manually through the solid-phase method using Fmoc chemistry (orthogonal base-labile protecting group) on Rink Amide resin (Orpegen Peptide Chemicals GmbH, Heidelberg, Germany). The following amino acid derivatives were used: Fmoc-l-Arg(Pbf)-OH, Fmoc-l-Cys(Trt)-OH, Fmoc-Gly-OH, and Fmoc-l-Lys(Boc)-OH. The aliphatic acids (Merck, Darmstadt, Germany) of hexadecanoic acid (C16), tetradecanoic acid (C14), dodecanoic acid (C12), and decanoic acid (C10), were attached to the N-terminus. All Nα-Fmoc-protected amino acids, the coupling reagents, and the resin were obtained from Iris Biotech GmbH (Marktredwitz, Germany). All reactions were run using a Skiller 1 (Kamush^®^, Gdansk, Poland) device to increase the efficiency of synthesis though the heating of the reaction vessel. The coupling reaction was carried out through activation with DIC (*N*,*N*’-diisopropylcarbodiimide, Carbolution Chemicals GmbH, St. Ingbert, Germany) in DMF (*N*,*N*-dimethylformamide, Honeywell, Seelze, Germany). OxymaPure (Iris Biotech GmbH, Marktredwitz, Germany) was applied to suppress racemization. All reagents were used in a fourfold excess based on the resin (Fmoc-AA: DIC: OxymaPure, 1:1:1, mol/mol). Double Fmoc deprotection was accomplished in a 20% piperidine (Merck, Darmstadt, Germany) solution in DMF. Deprotection was performed at 60 °C for 4 min (2 min + 2 min), whereas the coupling steps were performed at 60 °C (50 °C with cysteine) for 15 min. Every step was preceded by rinsing the resin and running the chloranil test. The peptides were cleaved from the resin using one of the following mixtures: (A)—trifluoroacetic acid (TFA, Apollo Scientific, Denton, UK), 1,2-ethanedithiol (EDT, Merck, Darmstadt, Germany), triisopropylsilane (TIS, Acros Organics, Geel, Belgium), and water (92.5:2.5:2.5:2.5 *v*/*v*/*v*/*v*); (B)—TFA, TIS, and water (95:2.5:2.5 *v*/*v*/*v*). Mixture (A) was used with peptides containing a cysteine residue, and mixture (B) was used for the remaining peptides. Cleavage was accomplished within 1 h while the mixtures were being stirred. Lipopeptide dimers were obtained by intermolecular disulfide bridge formation through oxidation with iodine (Table 1. Peptides 1, 3, 6).

The crude peptide was lyophilized and subsequently purified by RP-HPLC. The purifications were carried out on a Phenomenex Gemini-NX C18 column (21.20 × 100 mm, 5.0 µm particle size, and 110 Å pore size). Acetonitrile and water, both containing 0.1% of TFA, were used as the mobile phase. UV detection at 214 nm was used. The crude peptides were eluted with a linear 20–80% acetonitrile gradient in deionized water over 60 min at a flow rate of 10.0 mL/min. The purity and identity of the peptides were confirmed with LC-MS analysis. A Waters Alliance e2695 RP-HPLC system (Waters, Milford, MA, USA) with Waters 2998 PDA (Waters, Milford, MA, USA) and Acquity QDA detectors (Empower^®^3 software, Database Version 7.21.00.00) was used. All analyses were carried out on a Phenomenex, Luna C18(2) column (3.0 × 100 mm, 5.0 µm particle size, 100 Å pore size). The samples were analyzed with a linear 20–80% acetonitrile gradient in deionized water over 10 min at a flow rate of 0.5 mL/min with UV detection at 214 nm. Both eluents contained 0.1% (*v*/*v*) of formic acid. The pure fractions (>95%, by HPLC analysis) were collected and lyophilized. The retention time and percentage of ACN (%) at which each peptide was eluted are included in Table 1 to evaluate the hydrophobicity of lipopeptides. The identity of all compounds was confirmed using mass spectrometry (Waters Alliance e2695 system with Acquity QDA detector, Waters, Milford, MA, USA) with electrospray ionization in positive ion mode (ESI-MS). In this mode, a proton is attached to basic moieties (amine groups, guanidine groups). The theoretical mass of the peptide was calculated and then expected ions (*m*/*z*—mass to charge ratio) were compared to measured *m*/*z*. The results are presented in Table 1, and mass spectra are presented in the Supplementary Materials.

Lipopeptide 9 was synthesized manually through the solid-phase method using standard Fmoc chemistry according to the protocol described in the previous work [38].

The molecular structures were drawn with ChemSketch 2012 freeware software Version 14.01 (Advanced Chemistry Development, Inc., Toronto, On, Canada, www.acdlabs.com, 2019) and are included in the Supplementary Materials (Figure S1–S9).

### 2.2. Bacterial Strains and Media

The reference strains of gram-positive and gram-negative bacteria (Table 2) were obtained from the Polish Collection of Microorganisms (Polish Academy of Science, Wroclaw, Poland). The microorganisms were cultured in Muller Hinton Broth II (MHB II, Biocorp, Warsaw, Poland), overnight, at 37 °C.

### 2.3. Antimicrobial Activities of Lipopeptides and Conventional Antimicrobials

#### 2.3.1. Minimum Inhibitory Concentration (MIC) Assay

The MICs of synthetic lipopeptides and conventional antimicrobials were determined using the broth microdilution method according to the Clinical and Laboratory Standard Institute (CLSI) guidelines on reference strains [39]. Bacterial suspension at inoculums of ca. 5 × 10^6^ CFU/mL were added to 96-well spherical bottom polystyrene plates (PS, Kartell, Noviglio, Italy) and exposed to peptides at increasing concentrations (1–512 μg/mL). The PS were incubated under aerobic conditions at 37 °C for 18 h. The MIC (μg/mL) was assumed as the lowest concentration of the compound that inhibited the growth of the microorganisms. All experiments were performed in triplicate and included the growth and sterility controls.

#### 2.3.2. Minimum Biofilm Formation Inhibitory Concentration (MBFIC) Assay

To determine the inhibitory effect of antimicrobials on the formation of biofilm on hydrophobic surfaces, the bacterial suspensions of strains (initial inoculum of ca. 5 × 10^8^ CFU/mL in MHB II medium supplemented with antimicrobials) were cultured on 96-well PS at 37 °C for 18 h under aerobic conditions. After incubation, the suspensions were aspirated, and the wells were rinsed three times with sterile phosphate buffer PBS (AppliChem, Darmstadt, Germany) in order to remove the planktonic cells. Resazurin (final concentration per sample = 0.005%) was applied as a cell viability reagent for reading the results. In contact with the living cells, the dye is metabolized by bacterial dehydrogenases, resulting in a reduction of blue resazurin to pink resorufin. All assays were performed in triplicate and included the growth controls (positive controls: bacterial suspensions without antimicrobials) and the sterility controls (negative controls: a sterile MBH medium). After 1h of incubation, a visual evaluation of results was made. The MBFIC was taken as the lowest concentration of the compound at which the level of bacterial metabolism (i.e. color of resazurin) was comparable with the negative control. In a preliminary study, it was established that the application of lipopeptides at a concentration found active from visual evaluation caused the reduction of bacterial metabolism to approximately 10% (or less) of the positive control once the results were evaluated with a spectrophotometer. After 1 h of incubation with the dye, absorbance was measured at 570 and 600 nm using a microplate reader (Thermo Fisher Scientific, Waltham, MA, USA). The metabolic activity of bacteria in the samples was measured according to the formula presented in the previous paper [40].

The MBFIC assay with CL was applied in order to access the influence of lipopeptides on the biofilm formation on hydrogel surfaces. The entire sterile CL (1-Day Acuvue Moist, containing Etafilcon A., purchased from Johnson & Johnson (Vision Care, Jacksonsville, FL, USA), were placed in 24-well PS (Orange Scientific, Braine-l’ Alleud, Belgium) and the MBFICs were determined according to the above protocol.

#### 2.3.3. Minimum Biofilm Eradication Concentration (MBEC) Assay

The biofilms of bacteria associated with the biofilm-related infections were cultured on 96-well flat bottom PS and on CL placed in 24-well plates. The bacteria at initial inoculums of 5 × 10^8^ CFU/mL in MHB II were added to the plates and incubated at 37 °C under aerobic conditions. After 24 h of incubation, all cultures were rinsed three times with PBS and fresh medium was added. Subsequently, the obtained biofilm cultures were exposed to solutions of antimicrobials applied at graded concentrations ranging from 1 to 512 μg/mL for 24 h at 37 °C. The anti-biofilm activity of antimicrobials was visualized by resazurin (final concentration per sample = 0.005%) in the same way as in the MBFIC assay. All experiments were performed in triplicate and included growth controls (positive control: bacterial suspension without antimicrobials) and sterility controls (negative controls: sterile MBH II medium). The MBEC was defined as the lowest concentration of the applied compound at which the reduction of resazurin (color change) was comparable to the negative controls.

## 3. Results

### 3.1. MIC of Lipopeptides

The lipopeptides exhibited various antimicrobial activities towards the bacteria bred as liquid cultures (Table 3). In general, short lipopeptides showed stronger antimicrobial activity towards gram-positive strains in comparison with gram-negative ones. The PM exhibited the highest resistance towards all the compounds. Its growth was inhibited once the peptides were applied at very high concentrations (256–512 μg/mL) or not inhibited at all (lipopeptides 1, 2, and 6).

The most active compounds in this assay were lipopeptides 1 and 9. The bacterial growth of gram-positive strains was inhibited after exposure to C_12_-KKC-NH_2_ and (C_10_)_2_-KKKK-NH_2_ at concentrations of 2–8 μg/mL and 4–16 μg/mL, respectively. Peptide 9 inhibited the growth of EC and PA once applied at a concentration of 16 μg/mL. Lipopeptide 1 exhibited the same activity against EC once its MIC against PA was 32 μg/mL.

### 3.2. MBFIC of Lipopeptides

The results of the performed assays revealed that the supplementation of MHB II with lipopeptides inhibits the formation of biofilms on both polystyrene and hydrogel surfaces. The inhibitory activity varied depending on the applied material and tested bacterial strains (Table 4). In general, it was stronger for gram-positive bacteria.

Similar to in the MIC assay, PM turned out to be the most resistant strain. The peptides were effective only when applied at the highest concentrations or not active at all. The use of compounds at high concentrations was necessary in order to inhibit the formation of biofilm from PA, which was more affected by peptides in the MIC assay. In this case, only lipopeptide 9 gave relatively satisfying results. Its application at a concentration of 64 μg/mL inhibited the formation of biofilm on both types of surfaces. EC was more sensitive in comparison to PA and PM. The formation of an EC biofilm was inhibited by peptides when applied at concentrations equal or a few times higher than its MIC values. The most effective was lipopeptide 7. The determined MBFIC values were 32 and 64 μg/mL on CL and PS, respectively. The concentration of 64 μg/mL was also sufficient to inhibit the formation of the EC biofilm by lipopeptide 8 on both types of surfaces, lipopeptides 3 and 9 on CL, and lipopeptide 5 on PS. In the majority of compounds, the MBFIC determined for EC on CL was equal or lower than the values obtained on PS. Only in the case of lipopeptide 5 was it easier to inhibit the EC biofilm formation on PS than on CL.

Among gram-positive strains, EF was the least sensitive to the applied peptides. Similar to the MIC assay, the highest activity was demonstrated by lipopeptide 1. The determined MBFIC values were 16 and 64 μg/mL for PS and CL, respectively. The concentration of 64 μg/mL was the MBFIC for EF for the majority of compounds on both types of surfaces. Lipopeptides 2 and 6 were the weakest antimicrobials against the EF biofilm formation, but the results obtained on PS and CL did not differ. The supplementation of MHB II with lower concentrations of lipopeptides prevented the biofilm formation by both staphylococci. The most active compounds (lipopeptides 4, 8, and 9) inhibited the formation of an SA biofilm at concentrations of 2–16 μg/mL. For SE, lipopeptides 1 and 9 (8–16 μg/mL) were the most active. Interestingly, the MBFIC values obtained for SA and SE were very similar, which is in contrast to the results of the MIC assay where SE was significantly more susceptible to antimicrobials. To inhibit the formation of biofilm from SE, it was necessary to apply peptides at concentrations a few times higher than the MIC values, while for SA, usually the MBFICs were equal or even lower than the obtained MICs.

Certain differences between the influence on the formation of biofilm on PS and CL were also observed. The supplementation with the majority of peptides resulted in a stronger inhibition of the formation of biofilm on PS (the MBFIC for CL was twice as high as for PS). Only for lipopeptides 3 and 6 was this opposite, while for lipopeptide 4, the same values were determined for PS and CL. For SA, the influence on the formation of biofilm on different surfaces varied even more. For lipopeptides 1, 5, and 7, the MBFICs obtained for PS and CL were equal. For lipopeptides 2, 8, and 9, the formation of biofilm on PS was more strongly affected by the supplementation with peptides, in contrast to lipopeptides 3, 4, and 6, which caused a stronger inhibition of the formation of biofilm on CL. It is worth noting that lipopeptides 4 and 6 demonstrated abilities to inhibit SA biofilm formation at concentrations a few times lower than the MIC.

### 3.3. MBEC of Lipopeptides

In the performed assays, the lipopeptides exhibited various antibiofilm activities depending on the surface and tested strain (Table 5). The obtained MBEC values demonstrate that lipopeptides were relatively effective antibiofilm agents against gram-positive bacteria and rather weak or ineffective agents towards gram-negative bacteria.

The majority of the tested compounds exhibited strong antimicrobial activities against 1-day-old biofilm formed by staphylococci on polystyrene surfaces. The most susceptible strain was SE. Its biofilm was eliminated by the majority of compounds applied at concentrations of 4–16 μg/mL. To eradicate the SA cultured on PS, higher concentrations of those compounds were needed (16–64 μg/mL). The biofilm of both strains cultured on CL was in general less susceptible to the peptides in comparison to the structures formed on PS. In some cases, the discrepancies were significant. The MBEC values obtained for SA and SE cultured on CL were 16 or even 32 times higher than those determined for PS, which were usually similar to the obtained MIC values. Some higher concentrations but still close to the MICs were necessary to eliminate the EF biofilm from PS and CL. For this strain, the differences between the resistance of structures cultured on PS and CL were not so significant. For lipopeptides 3, 6, and 8, the MBECs determined for PS and CL were equal. Lipopeptides 2, 5, and 7 were slightly more active towards bacteria cultured on CL, while for the remaining compounds, the MBECs for CL were four times higher than those determined for PS.

On both types of surface, the biofilms formed by gram-negative bacteria were more difficult to eradicate with peptides. To eliminate the EC biofilm with the most effective compounds (lipopeptides 5, 7, and 9), they had to be applied at concentrations of 64–128 μg/mL. PA was even more difficult to eliminate from the surfaces. From the nine tested peptides, six failed to eradicate of PA from the polystyrene surface. The remaining compounds (lipopeptides 3, 7, and 9) exhibited antibiofilm activities at very high concentrations. Interestingly, the PA biofilm formed on CL was more susceptible to the compounds. Lipopeptides 1, 5, and 9 demonstrated antibiofilm activities at 256 μg/mL. It is worth noting that for lipopeptide 8, the MBEC determined for CL was only 32 μg/mL, while the peptide was not active towards PA biofilm formed on PS at the range of tested concentrations. As in previous assays, PM was the most problematic strain. The PM cultured on CL turned out to be resistant to all the compounds, while three peptides eradicated PM from the PS once applied at a concentration of 512 μg/mL (lipopeptides 3 and 7) and 256 μg/mL (lipopeptide 8).

## 4. Discussion

The use of biomaterials highly improves therapeutic options and a patient’s quality of life, but it also creates the risk of infection development [10,38,41,42]. AMPs and their analogues, such short cationic lipopeptides, have been investigated as innovative treatment and prevention strategies to overcome biofilm-related infections [41,43,44,45,46,47,48,49].

The obtained compounds demonstrated antibacterial activity once tested against planktonic bacterial cultures, similar to previously reported short lipopeptides [37,38,50]. They were ineffective against PM, which was difficult to eradicate with peptides (and antibiotics) in previously performed studies [20,51,52]. PM can cause infections of the urinary tract, skin, subcutaneous tissue, and wounds. PM is known as a biofilm former, and an evident increase of PM resistance to broad-spectrum fluoroquinolones and cephalosporins has been observed [52]. In the previous study, mature PM biofilms were resistant to the majority of tested peptides (citropin 1.1, omiganan, pexiganan, and C_16_-RR-NH_2_) and antibiotics (ciprofloxacin, gentamycin, and neomycin). Only polymyxin B and C_16_-KK-NH_2_ eradicated the PM biofilm applied at a concentration of 512 μg/mL [20]. Similar to other peptides chosen for the previous study, the compounds demonstrated rather weak activity against the EC and PA biofilm. The higher activity against the PA biofilm was demonstrated by some conventional antimicrobials: Ciprofloxacin and gentamicin eliminated 3-day-old structures once applied at concentrations of 4 and 16 μg/mL, respectively, while chloramphenicol and neomycin were weak antibiofilm agents [20]. In the case of the EC biofilm, ciprofloxacin, was already effective at a concentration of 1 μg/mL [20]. In the later study, ciprofloxacin permanently eliminated the EC biofilm from PS at a concentration of 16 μg/mL [40]. Chloramphenicol, neomycin, and gentamycin showed rather weak activity against the EC biofilms [20]. The other study demonstrated that even highly active compounds like ciprofloxacin and fosfomycin failed to eradicate the PA and EC biofilms [53].

The lipopeptides presented much higher antibiofilm activities in the case of gram-positive bacteria. However, significant discrepancies between biofilms formed on PS and CL were noticed. Regarding the influence of peptides on biofilm formation, the determined active concentrations were usually similar for both materials. In the previous study, the conventional antimicrobials ciprofloxacin, chloramphenicol, and neomycin failed to permanently eradicate biofilm formed by EF. Some activity was observed after the application of antibiotics at concentrations of 64–256 μg/mL, but after their withdrawal, the bacteria repopulated almost completely [40]. The difficulties in the elimination of the EF biofilms with ciprofloxacin was previously reported by other groups [54]. In physiological conditions, EF colonizes the human gastrointestinal tract. Enterococci are common etiological factors of nosocomial catheter-associated infections and high rates of resistance to many antibiotics, including clindamycin, cephalosporins, and aminoglycosides are observed [55]. In view of the poor activity of conventional antibiotics against the EF biofilm, the results obtained for the lipopeptides are very promising.

The antistaphylococcal activities exhibited by the lipopeptides are compatible with our previous results, where the MBECs for SA determined for other peptides were 32–64 μg/mL, which were much better in comparison to those obtained for conventional antimicrobials [20,40].

In the previous study, the SA formed biofilms which were resistant to ciprofloxacin, chloramphenicol, gentamycin and neomycin. In this study, the AMPs exhibited stronger antibiofilm activities against SE than conventional compounds [20]. The antistaphylococcal activities of many of the lipopeptides were confirmed on larger sets of clinical SA strains [36,56,57,58,59]. These results are very valuable, considering the fact that staphylococci are one of the most frequent causes of biofilm-associated infections [15]. It is worth mentioning that the concentrations that were active against staphylococcal biofilms were usually only a few times higher than the MICs, while for conventional antimicrobials, concentrations at least 50–100 times higher than the MICs are needed to eradicate biofilms [20,36].

Besides the promising activities of lipopeptides against the SE and SA biofilms, a strong potential in biofilm prevention was demonstrated. In contrast to the results of MBEC assay, no evident difference or pattern towards the tested material was noticed.

The physicochemical properties of biomaterials are known to influence bacterial colonization, however, the exact correlation is difficult to determine as the system is very complex. The differences in adhesion and biofilm formation of SA, SE, EC, and PA on different orthopedic metal implant materials have been reported. Bacterial colonization occurred on all tested materials: highly cross-linked polyethylene, titanium, stainless steel, trabecular metal, and cobalt–chromium alloy. The last demonstrated the highest resistance to bacterial adherence [60]. In a study of microbial adherence and colonization of a polyspecies biofilm on differently processed titanium surfaces, a moderate influence of surface roughness on biofilm formation was demonstrated [61]. Park, et al. studied the effects of the surface roughness of a resin composite on biofilm formation from *Streptococcus mutans* and demonstrated that the topography of the surface may be important for biofilm formation [62]. Hydrophobic materials have been reported as more resistant to biofilm formation. Moreover, it is easier to remove bacteria from them in the case of colonization. However, it is impossible to find a simple correlation because there are too many variables that influence the formation and persistence of the biofilm [63,64]. In our study, the biofilm formed by staphylococci on the hydrophilic surface of CL was much more resistant to peptides in comparison to the structure on PS. On the other hand, there were no such evident discrepancies in the results obtained for tested materials when the influence of lipopeptides on biofilm formation was studied. This is a very complex issue and definitely worth further investigation in order to determine the mechanisms via which the lipopeptides inhibit the formation of biofilm. This would provide valuable information with regard to their potential application in the prevention of biofilm-related infections. The conducted research on AMPs has demonstrated that human cathelicidin LL-37 inhibited PA biofilm formation by interfering with the adhesion and quorum sensing. The peptide also reduced the attachment and development of the SE biofilms [23,65]. LL-37-derived peptides like OP-145, SAAP-145, SAAP-148, and SAAP-267 inhibited the formation of biofilm by SA isolated from the biofilm-associated infection [66,67,68]. The adhesion of streptoccoci in the oral cavity was inhibited by lactoferrin [69]. There are also reports on the successful application of AMP-modified materials. The first stages of biofilm formation by *Porphyromonas gingivalis* on a titanium surface were reduced once it was modified with lactoferricin [70]. The coated CL reduced the PA-induced acute red eye in a guinea pig model [71]. Moreover, the peptide has also been successfully tested in a human clinical trial [72,73]. The immobilization of many other AMPs, e.g. GZ3.27, GL13K, SESB2V, bacitracin, hLF1-11, chimeric peptides, and Mel-4 onto the different materials of glass, silicon, and titanium, proved to be a promising strategy to prevent bacterial biofilm formation [74]. The lipopeptide C_16_-KK-NH_2_ proved to be effective in the prevention of SA biofilm-associated infection in a rat model [36]. Its co-immobilization with DNase I on polydimethylosilaxane material resulted in an effective antibiofilm surface against SA and PA [75]. Due to the similarity in structure, positive effects are expected of the immobilization of our compounds onto biomaterials in the future.

The obtained compounds exhibit promising antimicrobial activities that are comparable with native AMPs. Due to the much shorter peptide chains, lower amounts of substrates and other materials for synthesis are needed so the costs are reduced significantly. Moreover, the lipopeptides were obtained with a novel, optimized, temperature-assisted method of synthesis which is less time-consuming and allows the production costs of active compounds to be reduced further. Considering the economic advantages and the results of microbiological assays, there is no doubt that the presented compounds have a strong antibiofilm potential and are worth further investigation regarding their application for preventing and fighting bacterial biofilm. Beyond the extension of microbiological studies, there are still numerous other issues to be evaluated in order to ensure patient safety, since a knowledge gap exists with regards to toxicity, absorbance, metabolism, and the elimination of short cationic lipopeptides.

## Figures and Tables

**Table 1:** Tested peptides.

Lipopeptide	Sequence	Average Mass (Da)	Net Charge	MS Analysis			HPLC Analysis	
				za	*m/z*b	*m/z*c	tR’ (min)	% ACN
1	C_12_-KKC-NH_2_*	1115.64	+4	1 2 3	1115.78 558.39 372.60	1115.96 558.91 373.18	3.10	38.6
2	C_12_-KR-NH_2_	483.70	+2	1 2	484.40 242.70	484.68 243.03	2.32	33.9
3	C_14_-KKC-NH_2_ *	1171.75	+4	1 2 3	1171.84 586.42 391.72	1172.18 586.79 391.77	3.88	43.3
4	C_14_-KR-NH_2_	511.75	+2	1 2	512.43 256.72	512.68 257.08	3.29	39.7
5	C_16_-KGK-NH_2_	568.84	+2	1 2	569.48 285.24	569.69 285.71	3.95	43.7
6	C_16_-KKC-NH_2_ *	1227.86	+4	1 2 3	1227.90 614.46 409.97	1228.21 614.95 410.91	4.63	47.8
7	C_16_-KKK-NH_2_	639.97	+3	1 2	640.55 320.78	640.90 321.09	2.98	37.9
8	C_16_-KR-NH_2_	539.81	+2	1 2	540.46 270.73	540.68 271.00	4.06	44.3
9	(C_10_)_2_-KKKK-NH_2_	838.23	+3	1 2 3	838.69 419.85 280.23	838.94 420.28 280.68	3.27	39.6

* dimers with disulfide bridge; a—Ion charge; b—Calculated mass to charge ratio; c—Measured mass to charge ratio; tR’—adjusted retention time (min).

**Table 2:** Reference bacterial strains.

Bacterial Group	Species	Number
Gram-positive	*Staphylococcus aureus*	ATCC 25923
	*Staphylococcus epidermidis*	ATCC 14990
	*Enterococcus faecalis*	ATCC 29212
Gram-negative	*Escherichia coli*	ATCC 25922
	*Pseudomonas aeruginosa*	ATCC 9029
	*Proteus mirabilis*	PCM 543

**Table 3:** Minimum inhibitory concentration (MIC) of the lipopeptides (μg/mL).

Ordinal Number	Lipopeptide	*S. aureus*	*S. epidermidis*	*E. faecalis*	*E. coli*	*P. aeruginosa*	*P. mirabilis*
1	C_12_-KKC-NH_2_	8	2	4	16	32	>512
2	C_12_-KR-NH_2_	64	32	64	512	512	>512
3	C_14_-KKC-NH_2_	32	4	16	64	128	512
4	C_14_-KR-NH_2_	32	4	32	32	128	512
5	C_16_-KGK-NH_2_	64	32	32	16	256	512
6	C_16_-KKC-NH_2_	256	128	256	256	256	>512
7	C_16_-KKK-NH_2_	64	16	16	32	64	256
8	C_16_-KR-NH_2_	32	8	64	64	128	512
9	(C_10_)_2_-KKKK-NH_2_	8	2	16	16	16	512

**Table 4:** Minimum biofilm formation inhibitory concentration (MBFIC) of the lipopeptides (μg/mL) for polystyrene plates (PS) and contact lenses (CL).

Ordinal Number	Lipopeptide	*S. aureus*	*S. epidermidis*	*E. faecalis*	*E. coli*	*P. aeruginosa*	*P. mirabilis*
		PS/CL	PS/CL	PS/CL	PS/CL	PS/CL	PS/CL
1	C_12_-KKC-NH_2_	32/32	8/16	16/64	128/128	512/128	>512/>512
2	C_12_-KR-NH_2_	64/128	16/32	512/512	512/128	512/512	>512/512
3	C_14_-KKC-NH_2_	64/32	64/32	256/64	256/64	512/512	512/512
4	C_14_-KR-NH_2_	16/2	32/32	128/64	128/128	256/128	512/>512
5	C_16_-KGK-NH_2_	32/32	32/64	64/64	64/128	512/256	>512/512
6	C_16_-KKC-NH_2_	256/64	256/64	256/256	512/128	512/256	>512/512
7	C_16_-KKK-NH_2_	32/32	8/32	64/64	64/32	512/512	512/256
8	C_16_-KR-NH_2_	8/16	16/32	64/64	64/64	512/128	512/512
9	(C_10_)_2_-KKKK-NH_2_	8/16	8/16	64/64	128/64	64/64	512/512

**Table 5:** Minimum biofilm eradication concentration (MBEC) of the lipopeptides (μg/mL) for PS and CL.

Ordinal Number	Lipopeptide	*S. aureus*	*S. epidermidis*	*E. faecalis*	*E. coli*	*P. aeruginosa*	*P. mirabilis*
		PS/CL	PS/CL	PS/CL	PS/CL	PS/CL	PS/CL
1	C_12_-KKC-NH_2_	32/256	16/128	32/128	256/256	>512/256	>512/>512
2	C_12_-KR-NH_2_	64/>512	16/256	256/128	256/512	>512/>512	>512/>512
3	C_14_-KKC-NH_2_	128/128	128/512	256/256	512/512	256/>512	512/>512
4	C_14_-KR-NH_2_	16/512	4/32	64/256	128/256	>512/>512	>512/>512
5	C_16_-KGK-NH_2_	32/512	8/128	64/32	128/64	>512/256	>512/>512
6	C_16_-KKC-NH_2_	256/512	256/256	512/512	512/512	>512/>512	>512/>512
7	C_16_-KKK-NH_2_	32/256	8/64	64/16	64/128	512/>512	512/>512
8	C_16_-KR-NH_2_	32/128	16/32	64/64	256/128	>512/32	256/>512
9	(C_10_)_2_-KKKK-NH_2_	64/128	16/64	32/128	64/128	512/256	>512/>512

## Supplementary Materials

The following are available online at https://www.mdpi.com/1999-4923/11/10/506/s1, Figure S1. Molecular structure of C_12_-KKC-NH_2_ dimer, Figure S2. Molecular structure of C_12_-KR-NH_2_, Figure S3. Molecular structure of C_14_-KKC-NH_2_ dimer, Figure S4. Molecular structure of C_14_-KR-NH_2_., Figure S5. Molecular structure of C_16_-KGK-NH_2_, Figure S6. Molecular structure of C_16_-KKC-NH_2_ dimer., Figure S7. Molecular structure of C_16_-KKK-NH_2_, Figure S8. Molecular structure of C_16_-KR-NH_2_, Figure S9. Molecular structure of (C_10_)_2_-KKKK-NH_2_.

## Abbreviations

ACN	Acetonitrile
AMPs	Antimicrobial peptides
Boc	tert-butyloxycarbonyl
ATCC	American Type Culture Collection
C	Cysteine residue
CL	Contact lenses
CLSI	Clinical and Laboratory Standards Institute
DCM	Dichloromethane
DIC	*N*,*N*′-diisopropylcarbodiimide
DMF	*N*,*N*-dimethylformamide
EC	*Escherichia coli*
EF	*Enterococcus faecalis*
ESI-MS	Electrospray-ionization mass spectrometry
Fmoc	9-Fluorenylmethoxycarbonyl group
G	Glycine residue
K	Lysine residue
KP	Klebsiella pneumoniae
MBEC	Minimum biofilm eradication concentration
MBFIC	Minimum biofilm formation inhibitory concentration
MIC	Minimum inhibitory concentration
PA	*Pseudomonas aeruginosa*
PBS	Phosphate-buffer saline
PM	*Proteus mirabilis*
PS	Polystyrene plates
R	Arginine residue
RP-HPLC	Reverse-phase high-performance liquid chromatography
SA	*Staphylococcus aureus*
SE	*Staphylococcus epidermidis*
SPPS	Solid-phase peptide synthesis
SV	Streptococcus viridans
TFA	Trifluoroacetic acid
TIS	Triisopropylsilane
