# Supplementary material for: Influence of Short Cationic Lipopeptides with Fatty Acids of Different Chain Lengths on Bacterial Biofilms Formed on Polystyrene and Hydrogel Surfaces
Source: Pharmaceutics. 2019 Oct 1;11(10):506. doi: 10.3390/pharmaceutics11100506 (PMC6835763; doi:10.3390/pharmaceutics11100506)
Supplement: Supplementary file 1 [file pharmaceutics-11-00506-s001.pdf]

pharmaceutics

Supplementary Mateials: Influence of Short Cationic Lipopeptides with Fatty Acids of Different Chain Lengths on Bacterial Biofilms Formed on Polystyrene and Hydrogel Surfaces

Malgorzata Anna Paduszynska, Magdalena Maciejewska, Damian Neubauer, Krzysztof Golacki, Magdalena Szymukowicz, Marta Bauer and Wojciech Kamysz

Molecular structures

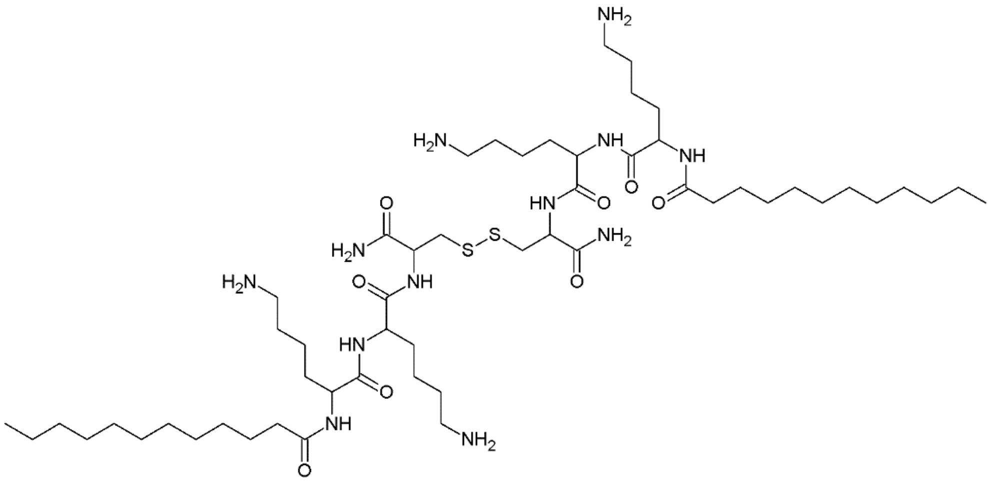

Figure S1. Molecular structure of C_{12}-KKC-NH_2 dimer.

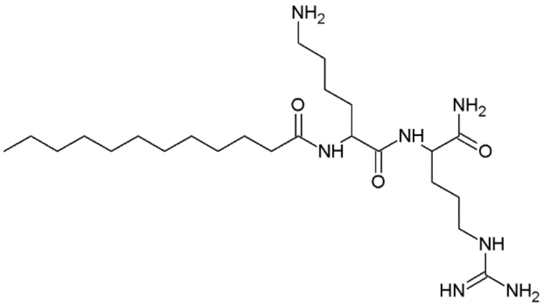

Figure S2. Molecular structure of C_{12}-KR-NH_2.

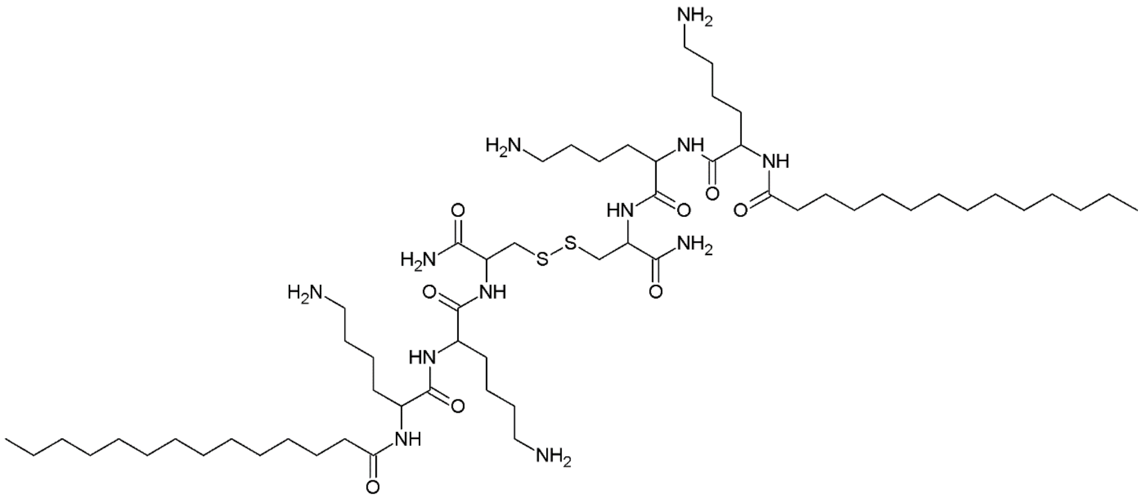

Figure S3. Molecular structure of C_{14}-KKC-NH_2 dimer.

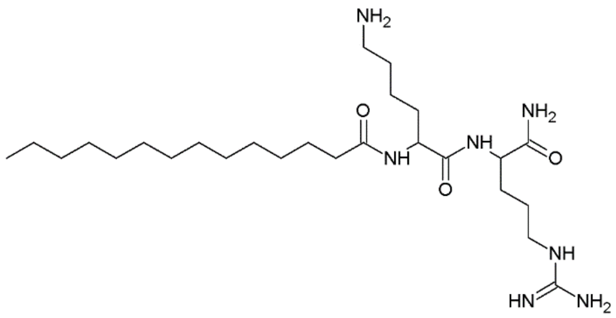

Figure S4. Molecular structure of C_{14}-KR-NH_2.

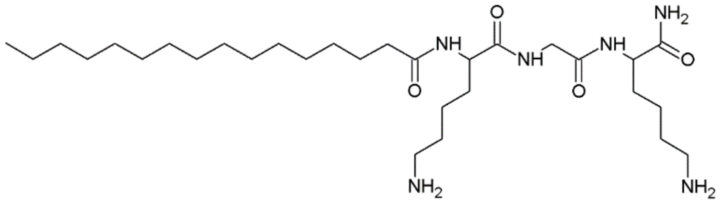

Figure S5. Molecular structure of C_{16}-KGK-NH_2.

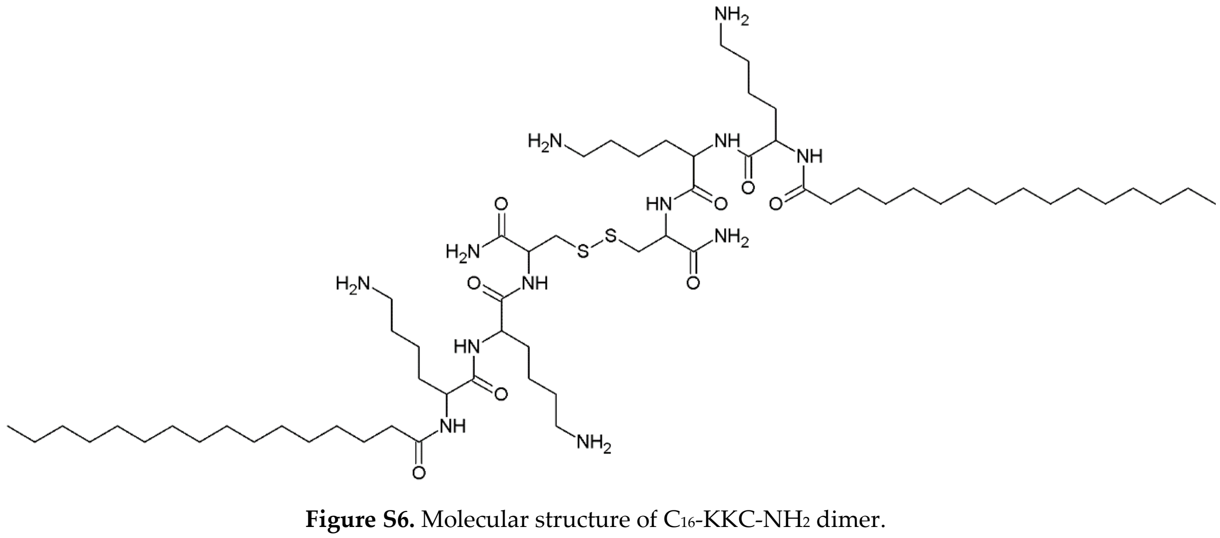

Figure S6. Molecular structure of C_{16}-KKC-NH_2 dimer.

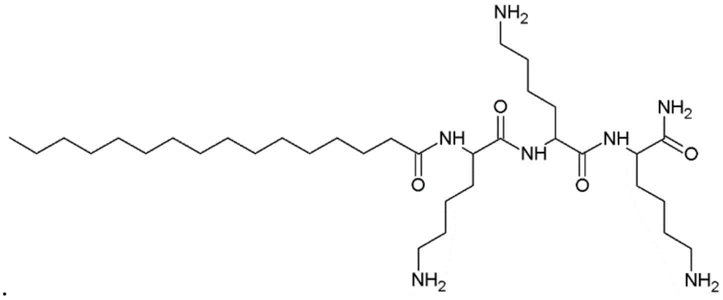

Figure S7. Molecular structure of C_{16}-KKK-NH_2.

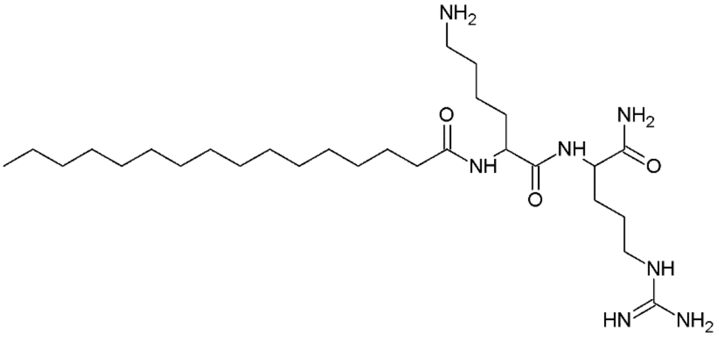

Figure S8. Molecular structure of C_{16}-KR-NH_2.

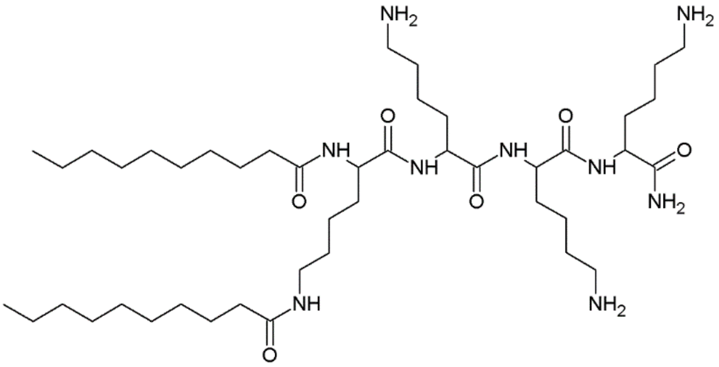

Figure S9. Molecular structure of $(C_{10})_2$-KKKK-NH_2.
